# Rictor Is Required for Early B Cell Development in Bone Marrow

**DOI:** 10.1371/journal.pone.0103970

**Published:** 2014-08-01

**Authors:** Yingchi Zhang, Tianyuan Hu, Chunlan Hua, Jie Gu, Liyan Zhang, Sha Hao, Haoyue Liang, Xiaomin Wang, Weili Wang, Jing Xu, Hanzhi Liu, Bin Liu, Tao Cheng, Weiping Yuan

**Affiliations:** 1 State Key Laboratory of Experimental Hematology, Institute of Hematology and Blood Diseases Hospital, Chinese Academy of Medical Sciences & Peking Union Medical College, Tianjin, China; 2 Center for Stem Cell Medicine, Chinese Academy of Medical Sciences, Beijing, China; 3 307-Ivy Translational Medicine Center, Laboratory of Oncology, Affiliated Hospital of Academy of Military Medical Sciences, Beijing, China; Emory University, United States of America

## Abstract

The development of early B cells, which are generated from hematopoietic stem cells (HSCs) in a series of well-characterized stages in bone marrow (BM), represents a paradigm for terminal differentiation processes. Akt is primarily regulated by phosphorylation at Thr308 by PDK1 and at Ser473 by mTORC2, and Akt signaling plays a key role in hematopoiesis. However, the role of mTORC2 in the development of early B cells remains poorly understood. In this study, we investigated the functional role of mTORC2 by specifically deleting an integral component, Rictor, in a hematopoietic system. We demonstrated that the deletion of Rictor induced an aberrant increase in the FoxO1 and Rag-1 proteins in BM B cells and that this increase was accompanied by a significant decrease in the abundance of B cells in the peripheral blood (PB) and the spleen, suggesting impaired development of early B cells in adult mouse BM. A BM transplantation assay revealed that the B cell differentiation defect induced by Rictor deletion was not affected by the BM microenvironment, thus indicating a cell-intrinsic mechanism. Furthermore, the knockdown of FoxO1 in Rictor-deleted HSCs and hematopoietic progenitor cells (HPCs) promoted the maturation of B cells in the BM of recipient mice. In addition, we revealed that treatment with rapamycin (an mTORC1 inhibitor) aggravated the deficiency in B cell development in the PB and BM. Taken together, our results provide further evidence that Rictor regulates the development of early B cells in a cell-intrinsic manner by modifying the expression of FoxO1 and Rag-1.

## Introduction

Adult B lymphocytes develop in bone marrow (BM), where B lymphoid-specified progenies are gradually generated from hematopoietic stem cells (HSCs) and lose the potential to differentiate into other blood lineage cells [1]. Early B cell development in BM is a highly ordered process involving the rearrangement of heavy-chain and light-chain gene segments. Pro-B cells in BM that are committed to the B lineage undergo V-DJ recombination at the immunoglobulin (Ig) heavy-chain locus, and cells with functional heavy chains are selected via the pre-B cell receptor (pre-BCR) to generate pre-B cells. In this process, the interleukin-7 receptor (IL-7R) cooperates with recombination-activating gene 1 (Rag-1) and Rag-2 proteins to catalyze V-DJ recombination [2]. The majority of Ig light-chain rearrangements occur in pre-B cells. Cells that undergo productive light-chain rearrangements yield immature B cell receptor-positive (BCR^+^) B cells [3]. To develop further, these immature B cells leave the BM and enter peripheral lymphoid tissues, such as the spleen, where transitional B cells differentiate into functionally distinct B cell subpopulations. These subpopulations include follicular and marginal zone B cells that can subsequently respond to T cell-dependent and T cell-independent antigens, respectively [4], [5].

The development of early B cells in BM represents a paradigm for a terminal differentiation process involving the step-wise conversion of a multipotent stem cell into a highly specialized cell type. Previous studies have demonstrated a key role for phosphatidylinositol 3-kinase (PI3K) signaling in this process [6], [7], [8], [9]. PI3Ks form a family of lipid kinase enzymes that generate 3′-phosphorylated phosphoinositides. Class I PI3Ks use PtdIns-4,5-bisphosphate (PIP2) as a substrate to produce PtdIns-3,4,5-trisphosphate (PIP3) [10] and to integrate several signaling events that are controlled by Syk, which phosphorylates several key proteins, including B cell adaptor for phosphoinositide 3-kinase (BCAP) and CD19. These proteins contribute to the PI3K activation initiated by the pre-BCR or the BCR [11].

The serine/threonine kinases Akt and phosphoinositide-dependent kinase-1 (PDK-1) are activated by PI3K in all cells, including B cells [12].The Akt family is expressed in three distinct isoforms:Akt1, Akt2, and Akt3 [13]. All of these proteins share similar structures and functions and regulate cell survival and proliferation by activating multiple downstream signaling pathways. All three Akt isoforms are expressed in B lineage cells, and their functions appear to be partially redundant. Recent observations have shown that Akt1 and Akt2 promote peripheral B cell maturation and survival [14]. The forkhead box O (FoxO) transcription factors (FoxO1, FoxO3a, FoxO4, and FoxO6) are downstream of Akt signaling and are particularly important for B cell development [15], [16].The Akt-mediated phosphorylation of FoxOs can suppress the transcriptional activity of these factors and causes their nuclear export and degradation. FoxO1 is an essential component of a transcription factor network in pro-B cells that also includes Transcription factor 3 (TCF3 or E2A)and early B-cell factor 1 (EBF1) [17]. FoxO1 functions with E2A and EBF1 to induce transcription of the *Pax5* gene to drive B cell commitment. FoxO1 is essential for B cell development, as FoxO1 knockout studies have demonstrated. Using mice with a conditional allele of *FoxO1*, Rickert and his colleagues analyzed the function of FoxO1 at various stages using various Cre mouse strains. FoxO1 deletion at an early stage using *Mb1*-Cre caused a partial block at the pro-B cell stage that could be attributed to impaired IL-7R expression. Deletion in late pro-B cells using *Cd19*-Cre caused a block at the pre-B cell stage because of reduced expression of Rag-1 and Rag-2 [16].

Cell growth and proliferation are regulated by multiple signals, including growth factors, nutrients, and stress. One of the key components regulating these signal transduction pathways is mammalian target of rapamycin (mTOR). In mammals, mTOR is the key component of two distinct multi-protein complexes, mTOR complex 1 (mTORC1) and 2 (mTORC2). mTORC1 is sensitive to rapamycin and is composed of mTOR (catalytic subunit), mammalian lethal with SEC13 protein 8 (mLST8/GβL), 40 kDa Pro-rich Akt substrate (PRAS40), the regulatory associated protein of mTOR (Raptor), and DEP domain-containing mTOR-interacting protein (DEPTOR). mTORC1 controls protein synthesis and cell growth by regulating the phosphorylation of the downstream translation regulator eIF-4E-binding protein 1 (4E-BP1) and the protein kinase AGC member S6 kinase 1 (S6K1). mTORC2 is insensitive to rapamycin and includes mTOR, rapamycin-insensitive companion of mTOR (Rictor), mLST8, mammalian stress-activated map kinase-interacting protein 1 (mSin1), protein observed with Rictor 1 (Protor-1), and Protor-2 [18]. mTORC2 modulates cell survival in response to growth factors by regulating the phosphorylation of Akt, serum/glucocorticoid regulated kinase 1 (SGK1), and protein kinase Ca (PKCa) [19]. A recent report demonstrated that mTORC1 is activated in response to different B cell stimuli through distinct signaling mechanisms [20]. Benhamron and his colleagues reported that the activation of mTORC1 in B lymphocytes impaired B cell maturation and reduced marginal-zone B cells [21]. Recent studies showed that the deletion of *Raptor*, but not *Rictor*, impaired the function of normal HSCs. In contrast, *Rictor* deletion prevented leukemogenesis and HSC depletion after *phosphatase and tensinhomologue (Pten)* deletion in adult mice. These studies also indicated that deletion of *Raptor* or *Rictor* would induce a defect in B-cell numbers [22], [23]. However, the studies did not explore the role of mTORC1 and mTORC2 in lymphoid development. Furthermore, the role of mTORC2 in B cells, particularly early B cell development in BM, is still not completely understood. In this study, using *Rictor* conditional knockout mice, we showed that *Rictor* deletion led to a decrease in the abundance of B cells in the peripheral blood (PB) and the spleen and impaired early B cell development in BM. Rictor-deficient B cells exhibited an aberrant increase in FoxO1 and Rag-1, which may have led to the observed defect in early B cell development in BM. Furthermore, rapamycin treatment aggravated the *Rictor* deletion-induced defect in B cells via the inhibition of mTORC1 activity.

## Materials and Methods

### Mice and genotyping

Rictor^fl/fl^ mice were generously provided by Dr. Mark A. Magnuson [24] and were backcrossed for 10 generations onto the C57BL/6 background. Transgenic mice expressing Cre recombinase under the control of the Mx1 gene (Mx1 Cre) were purchased from the Jackson Laboratory. The transgene was crossed into Rictor^fl/fl^ mice to generate control (Mx-1 Cre^−^ Rictor^fl/fl^) and *Rictor* conditional knockout (Mx-1 Cre^+^Rictor^fl/fl^) mice. Genotyping was performed with PCR using DNA extracted from ear or tail biopsies. The following primers were used for genotyping: Rictor^fl^, 5′-ACTGAATATGTTCATGGTTGTG and 5′-GAAGTTATTCAGATGGCCCAGC; Mx-1 Cre, 5′- CTCTGGCCATCTGCTGATCC and 5′-CGCCGCATAACCAGTGAAAC. All of the animal protocols were approved by the Institutional Animal Care and Use Committee (IACUC), Institute of Hematology and Blood Diseases Hospital, CAMS/PUMC. All surgery was performed under sodium pentobarbital anesthesia, and all efforts were made to minimize suffering.

### pIpC and rapamycin treatment

Polyinosinic-polycytidylic acid (pIpC; Amersham) was dissolved in PBS at 2.5 mg/ml. The mice received 5 mg/kg of pIpC for a total of four i.p. injections once every other day. The deletion of *Rictor* exon 3 was assessed using a 7500 real-time PCR system (Applied Biosystems) with the primers 5′-TGAAACCCGTCAATATGGCG and 5′-CAGATGGCCCAGCTTTCTCA. Rapamycin (LC lab) was reconstituted in absolute ethanol at 10 mg/ml and diluted in 5% Tween-80 (Sigma-Aldrich) and 5% PEG-400(Sigma-Aldrich). The recipient mice transplanted with Rictor-deleted and control BMNCs received 4 mg/kg rapamycin by i.p. injection every other day for 3 months.

### Flow cytometry

Blood was drawn from the tail vein and stained with antibodies (Abs). After staining, red blood cells were lysed using BD FACS lysing solution (BD Biosciences). Spleen cells and BM isolated from the femur, tibia, and iliac were suspended in staining buffer (PBS with 2%FBS and 2 mM EDTA) with the following Abs purchased from eBioscience and BD Biosciences: CD3, CD4, CD8, B220, Mac-1, c-Kit, Sca-1, CD34, Flk2, CD43, CD19, AA4.1, and IgM. The lineage cocktail was composed of the following Abs: CD3, CD4, CD8, B220, Ter-119, Mac-1, and Gr-1. All Abs were directly conjugated to FITC, PE, Percp-Cy5.5, APC, PE-Cy7, and APC-Cy7. Streptavidin conjugates were purchased from BD Biosciences. After immunostaining, the cells were suspended and maintained at 4°C prior to FACS analysis. For cell-cycle analysis, BM cells from the control and Rictor-deleted mice were labeled with B220, CD19, CD43, AA4.1, and IgM antibodies, fixed and permeabilized using a BD IntraSure Kit (BD Bioscience), and then intracellularly stained with an antibody against Ki67 and with Hoechst 33342 (BD Bioscience). BM cells from the control and Rictor-deleted mice labeled with committed B cell-specific antibodies were stained with Annexin V and 7-AAD (BD Bioscience) for the apoptosis assay. The cell data were acquired using an LSRII flow cytometer, and cell-sorting data were acquired using a FACS Aria-III system (Becton Dickson). Data analysis was performed using FlowJo.

### RNA purification and real-time PCR

Total RNA was extracted using an RNeasy Mini Kit (QIAGEN). Reverse transcription was performed using Oligo(dT)_18_, 2×TS Reaction Mix, and TransScript RT/RI Enzyme Mix (Transgen). Real-time PCR was conducted with a FastStart Universal SYBR Green Master (Roche), 0.4 µM of specific forward and reverse primers, and normalized cDNA. The parameters for the PCR thermal cycling consisted of 40 cycles of 15 s at 95°C and 60 s at 60°C. The measured transcript copy numbers were normalized using GAPDH as a reference. The fold changes were calculated according to the ΔΔCT method. The following primers were used: *FoxO1*, 5′-GGGTCCCACAGCAACGATG and 5′-CGAGGACGAAATGTACTCCAGTT; *IL-7R*, 5′-AAGTGGAAATGCCCAGGAT and 5′-TTGACTTCCATCCACTTCCA; *Pax5*, 5′-TCCCAGATGTAGTCCGCCAAA and 5′-TCCTGTCTCATAATACCTGC-CAA; *Rag-1*, 5′-TGCAGACATTCTAGCACTCTGG and 5′-ACATCTGCCTTCAC-GTCGAT; *Rag-2*, 5′-AGGCTGGCCTAAGAGATCCTG and 5′-GCCTTTGTA-TGAGCAAGTAGCTG; *GAPDH*, 5′-GCCCACTTGAAGGGTGGAGC and 5′-CATGAGCCCTTCCACAATG.

### Western blotting analysis and immunofluorescence staining

BM cells from control and Rictor-deficient mice were stained with anti-mouse B220 microbeads and enriched using a separation column (Miltenyi Biotec) according to the manufacturer's instruction. After sorting, the B cells were washed twice with ice-cold PBS and then lysed using lysis buffer. Total cell lysate (20 µg) was heated at 95°C for 5 min in sample buffer, subjected to PAGE, and then electrotransferred onto nitrocellulose membranes. Detection was performed using HRP-conjugated secondary antibodies and enhanced chemiluminescence (ECL) reagent. Rictor antibody was obtained from Bethyl Laboratories, Inc. Akt, Akt-Ser473, FoxO1-Ser256, FoxO1, Rag-1, IL-7R and Actin antibodies were purchased from Cell Signaling Technology. For the immunofluorescence procedure, B cells from control and Rictor-deleted mice were sorted with MACS according to the manufacturer's instruction. The cells were then fixed with 4% paraformaldehyde solution, permeabilized with 0.25% Triton X-100, washed twice with PBS and blocked with 1% BSA for 30 min at room temperature. The samples were then incubated with FoxO1 antibody diluted 1∶100 in 1% BSA for 2 h at room temperature. Primary antibodies were detected using secondary antibody (donkey anti-rabbit Alexa Fluor 647, Invitrogen) diluted 1∶200 with1% BSA for 1 h at room temperature. The samples were then washed twice. After 5 ug/ml DAPI was added, the samples were examined under an Axio Imager Z2 fluorescence microscope (ZEISS).

### Viral production and transduction of Lin^−^c-Kit^+^ cells

pGIPZ lentiviral mouse FoxO1 shRNAs were obtained from Open Biosystems, and the SF-LV-shRNA-EGFP vector was generously provided by Dr. Rudolph [25]. FoxO1 shRNA was cut from the pGIPZ vector by XhoI and MluI and then cloned into an SF-LV lentiviral vector. The plasmids SF-LV-shFoxO1-EGFP, pSPAX2 and pMD2.G were co-transfected into the packaging cell line 293T using Lipofectamine 2000 (Invitrogen) to generate the virus. Virus supernatants were harvested 48 and 72 h after transfection. Lin^−^c-Kit^+^ cells from the BM of Rictor-deleted mice were enriched using a Lineage Cell Depletion Kit and c-Kit (CD117) magnetic beads (MiltenyiBiotec) according to the manufacturer's protocol. The Lin^−^c-Kit^+^ cells were then transduced with viruses, and the transduction efficiency was measured using flow cytometry. BM mononucleated cells (BMMNCs) from 8-week-old B6.SJL mice (CD45.1^+^) with virus-transduced Lin^−^c-Kit^+^ cells were transplanted into lethally irradiated (9.6 Gy) B6.SJL mice.

### Statistical analysis

Student's t test was used for comparisons between 2 groups, and ANOVA was used for multiple groups. All results represent the average of at least three independent experiments and are expressed as the mean ± SD. P<0.05 was considered significant.

## Results

### Rictor deletion caused a decrease in the abundance of B cells in the PB and spleen

**Figure 1:**
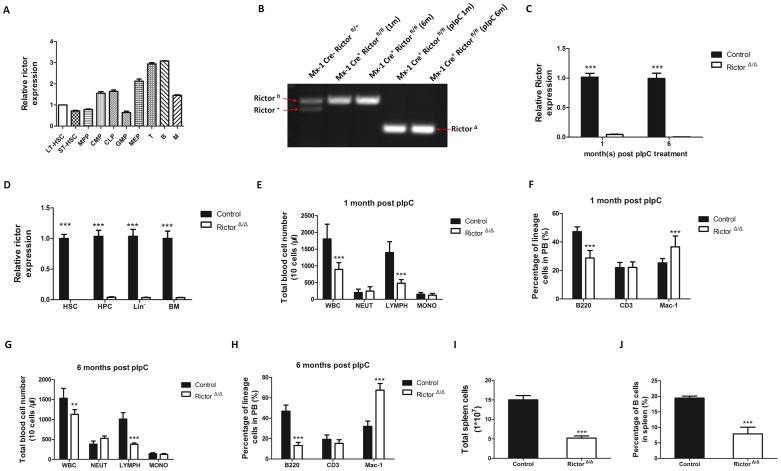
Rictor deletion leads to a decrease in B cells in the PB and spleen. (A) 1×10^5^ LT-HSC, ST-HSC, MPP, CLP, CMP, GMP, MEP, T, B and myeloid cells were sorted with FACS for real-time PCR. The data shown have been normalized to GAPDH. (B) *Rictor* excision in BM cells. BMMNCs were sorted fromMx-1 Cre^+^ Rictor^fl/fl^ micethat did or did not undergo pIpC treatment, and PCR was performed using two different sets of primers to detect *Rictor* in WT (*Rictor* ^+^), conditional (*Rictor* ^fl^) and deleted (*Rictor* ^Δ^) alleles. (C) Rictor is efficiently deleted in the BM of Mx-1 Cre^+^ Rictor^fl/fl^ mice with pIpC treatment for 1 month or 6 months. One or 6 months after pIpC treatment, 1×10^6^BMMNCs were isolated from Mx-1 Cre^+^Rictor^fl/fl^mice for real-time PCR. (D) Rictor was efficiently deleted in HSCs and HPCs. One month after pIpC treatment, 1×10^5^HSC, HPC and Lin^−^ cells were sorted with FACS for real-time PCR. The data shown were normalized to GAPDH. (E) and (G) The PB counts of control and Rictor-deleted mice1 and 6 months after pIpC treatment (n = 10). (F) and (H) The percentage of T (CD3^+^), B (B220^+^) and myeloid cells (Mac-1^+^) in PB were analyzed after 1 and 6 months of pIpC treatment by flow cytometry (n = 10). (I) The total number of spleen cells in the control and Rictor-deleted mice were counted using a hemocytometer (n = 5). (J) The percentage of B cells in the spleen was analyzed by flow cytometry (n = 5). The flow cytometry and hemocytometry measurements were performed in triplicate(*: P<0.05; **: P<0.01; ***: P<0.001).

We first assessed the mouse *Rictor* gene expression in purified long-term hematopoietic stem cells (LT-HSCs: CD34^−^Flt3^−^Lin^−^Sca1^+^c-Kit^+^), short-term hematopoietic stem cells (ST-HSCs: CD34^+^Flt3^−^Lin^−^Sca1^+^c-Kit^+^), multipotent progenitor cells (MPPs: CD34^+^Flt3^+^Lin^−^Sca1^+^c-Kit^+^), common lymphoid progenitors (CLPs: Lin^−^Sca-1^low^c-Kit^low^IL-7R^−^), common myeloid progenitors (CMPs: CD34^+^FcγRII/III^−^Lin^−^Sca1^−^c-Kit^+^), granulocyte-macrophage progenitors (GMPs: CD34^+^FcγRII/III^+^Lin^−^Sca1^−^c-Kit^+^), megakaryocyte-erythrocyte progenitors (MEPs: CD34^−^FcγRII/III^−^Lin^−^Sca1^−^c-Kit^+^), T cells (CD3^+^), B cells (B220^+^), and myeloid cells (Mac-1^+^) using quantitative real-time PCR. The T cells and B cells showed a higher expression of *Rictor* in these populations (Figure 1A), indicating that Rictor may play a pivotal role in these cells. To further address the biological function of Rictor in hematopoiesis, we deleted *Rictor* in the hematopoietic system in Rictor-floxed mice that expressed Mx1-Cre. At 8 weeks of age, the Mx-1 Cre^−^Rictor^fl/fl^, and Mx-1 Cre^+^ Rictor^fl/fl^ (abbreviated hereafter as control and Rictor^Δ/Δ^ in the figures) mice were treated with pIpC every other day for 1 week to delete the *Rictor* gene. As Figure 1B–D shows, the *Rictor* gene was almost completely deleted in the BMMNCs, HSCs (Lin^−^Sca-1^+^c-Kit^+^), HPCs (Lin^−^Sca-1^−^c-Kit^+^), and lineage-negative (Lin^−^) cells after the pIpC treatment. The quantity and percentage of multilineage cells in the PB were monitored for up to 6 months after the pIpC treatment by cell counting and flow cytometry. We found that the Rictor-deleted mice exhibited a significant decrease in the amount of whole blood cells and lymphoid blood cells 1 or 6 months after the pIpC treatment (Figure 1E and 1F). In addition, the percentage of B cells was lower in the *Rictor*-deleted mice than in the controls (Figure 1G and 1H). Furthermore, the quantity and percentage of B cells in the spleen were significantly lower after *Rictor* deletion compared with the control mice (Figure 1I and 1J).

### Rictor deletion impaired early B cell development in BM

**Figure 2:**
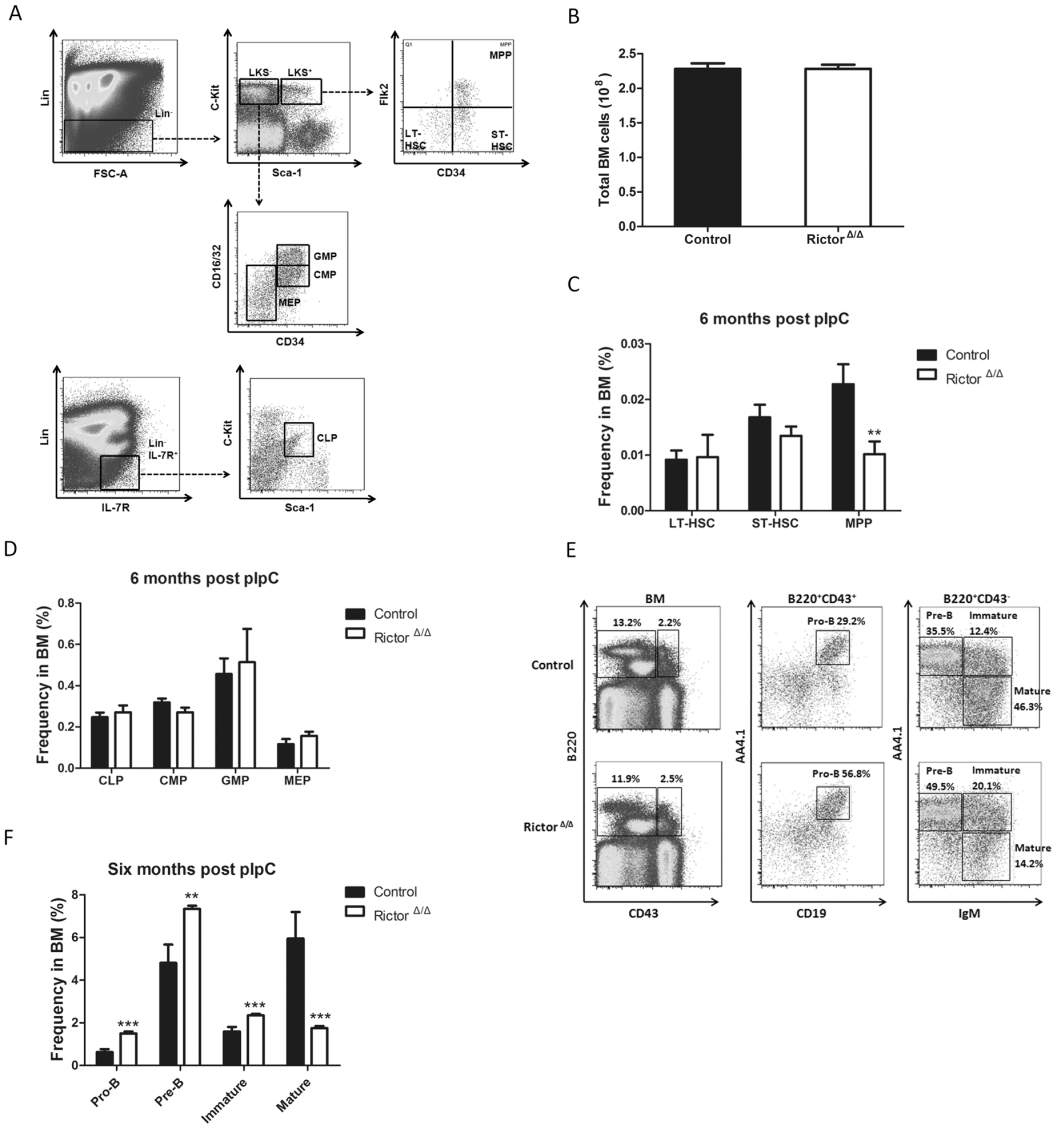
Rictor deletion impaired early B cell development in BM. (A) Representative flow cytometry profile of hematopoietic stem cells and progenitor cells based on the indicated surface marker. (B) Total BM cell count. (C) and (D) The percentages of stem and progenitor cells were analyzed by flow cytometry after 6 months of pIpC treatment (n = 5). (E) The representative flow cytometry profile for committed B cells in BM. (F) The percentage of committed B cells in BM after 6 months of pIpC treatment (n = 5). All of these experiments were performed in triplicate (*: P<0.05, **: P<0.01, ***: P<0.001).

Next, we examined whether the deletion of *Rictor* would reduce the number of whole BM cells. We found that the total number of BM cells in the *Rictor*-deleted mice was similar to that of the control mice (Figure 2B). To further investigate whether *Rictor* deletion impairs early hematopoietic cell development, we examined the percentage of LT-HSCs, ST-HSCs, and MPPs using flow cytometry. As Figure 2C shows, *Rictor* deletion did not change the HSC composition. Subsequently, we examined whether *Rictor* deficiency affected HPCs. We found that the percentages of CLPs, CMPs, GMPs, and MEPs in the Rictor-deleted mice were similar to those in the control mice (Figure 2D). Furthermore, we examined early B cell development in the Rictor-deleted mice via examining the expression of the cell surface markers B220, CD43, CD19, IgM and AA4.1 [14]. As shown in Figure 2F, we found that the percentages of pro-B (B220^+^CD43^+^CD19^+^AA4.1^+^), pre-B (B220^+^CD43^low^IgM^−^AA4.1^+^), and immature B cells (B220^+^CD43^−^IgM^+^AA4.1^+^) in the BM were dramatically increased in the Rictor-deleted mice compared with the control, whereas the percentage of mature B cells (B220^+^CD43^−^IgM^+^AA4.1^−^) in the BM was significantly decreased. These data indicate that *Rictor* deletion led to a defect in early B cell development in BM.

### Impaired B cell development in Rictor-deficient mice resulted from a cell-intrinsic defect

**Figure 3:**
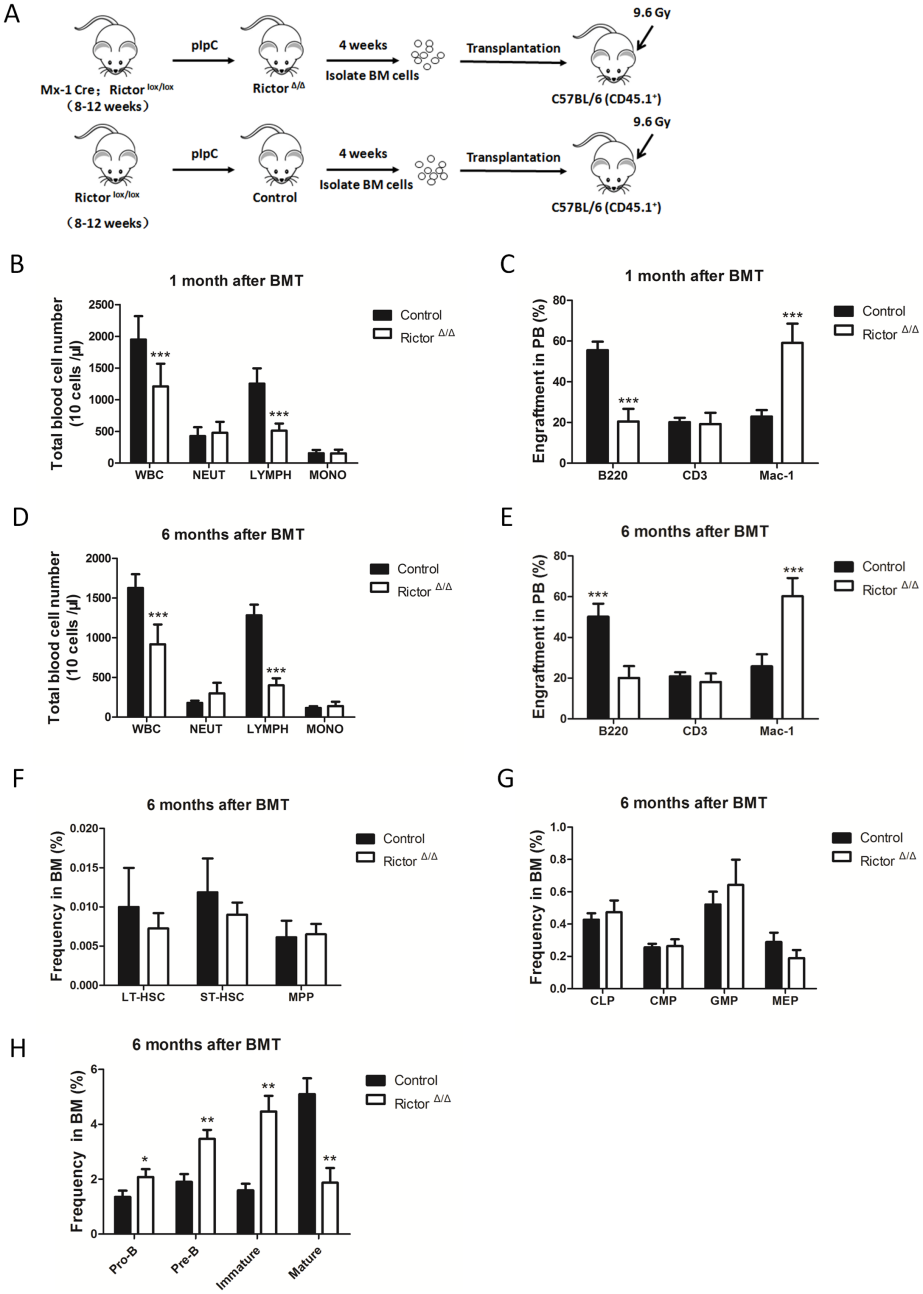
The impaired B cell development in Rictor-deficient mice resulted from a cell-intrinsic defect. (A) A schematic diagram of the experimental design. (B) and (D) PB cells were counted1 and 6 months after transplantation (n = 10). (C) and (E) The percentages of multilineage cells in the PB were analyzed by flow cytometry 1 and 6 months after transplantation (n = 10). (F), (G), and (H) The percentages of HSPCs and committed B cells in BM were examined 6 months after transplantation (n = 5). All of these experiments were performed in triplicate (*: P<0.05; **: P<0.01; ***: P<0.001).

Because the pIpC treatment induced *Rictor* deletion in all Mx-1-expressing tissues of the Mx-1 Cre^+^ Rictor^fl/fl^ mice [26], we used a transplantation approach to determine whether the impaired B cell development was intrinsic or extrinsic to the cell during early B cell development in the *Rictor*-deleted mice. Lethally irradiated (9.6 Gy) congenic recipient mice (CD45.1) received a transplant of 1×10^6^ BMMNCs from control and *Rictor*-deleted mice (donor, CD45.2), and FACS analysis was performed to evaluate the repopulation of donor cells in the recipient mice 1 and 6 months after transplantation (Figure 3A). The results showed that the percentage of B cells in the PB of the *Rictor*-deletion recipient mice was significant lower than that in the control (Figure 3B–3E). Subsequently, we examined the differentiation of donor cells in BM and found that the percentages of HSCs and HPCs in the BM of the *Rictor*-deletion recipient mice were similar to those in the control (Figure 3F and 3G). The percentages of pro-B, pre-B, and immature B cells were significantly higher in the BM of the *Rictor*-deletion recipient mice than those in the control, whereas the percentage of mature B cells was lower in the BM of the *Rictor*-deletion recipient mice compared with the control (Figure 3H). These data suggest that the defect in the B cell development of *Rictor*-deleted mice was independent of the hematopoietic environment and that the defect in the B cell differentiation of *Rictor*-deleted mice was mediated by a cell-intrinsic mechanism.

### Rictor deficiency altered the cell cycle of mature B cells in BM

**Figure 4:**
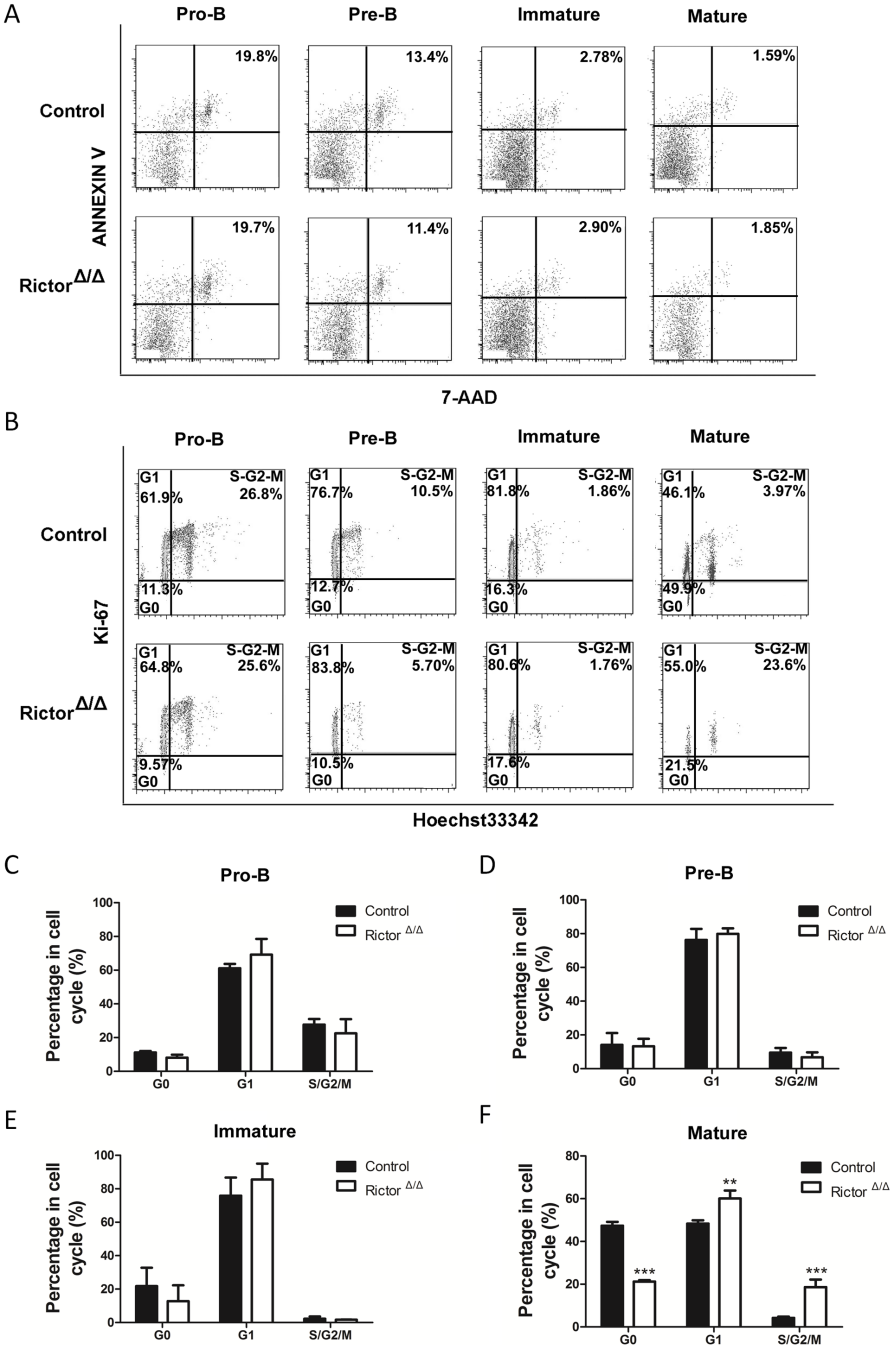
Rictor deficiency altered the cell cycle of the mature B cells in BM. (**A**) Flow cytometry analysis of committed B cell apoptosis (n = 5). (B) The representative cell cycle of committed B cells from control and *Rictor*-deleted mice (n = 5). (C), (D), (E), and (F) Cell cycle analysis of pro-B, pre-B, immature B, and mature B cells (n = 5). All of these experiments were performed in triplicate (*: P<0.05; **: P<0.01; ***: P<0.001).

To explore the mechanism of the early B cell development defect in the Rictor-deleted mice, we analyzed the apoptosis status of pre-B, pro-B, immature B, and mature B cells using flow cytometry. As shown in Figure 4A, the apoptosis of the B cells in the BM did not significantly differ between the *Rictor*-deleted mice and the control mice. We then examined whether there was any difference in the cell cycle status in the pre-B, pro-B, immature B, and mature B cells between the Rictor-deleted mice and the control mice. We found that *Rictor* deletion affected the cell cycle of the mature B cells but not that of the pro-B, pre-B, and immature B cells. There were fewer mature B cells in the G0 phase and more mature cells in the G1 and S/G2/M phases in the Rictor-deleted mice compared with the control mice. This finding was inconsistent with the observation that *Rictor* deletion led to a reduction in mature B cells in the BM because more mature B cells in the G1 and S/G2/M phases would produce more mature B cells. These results suggested that *Rictor* deletion induced other defects that caused the increased abundance of pro-B, pre-B, and immature B cells and the decreased abundance of the mature B cells in BM.

### Rictor-deficient B cells exhibited increased expression of FoxO1 and Rag-1

**Figure 5:**
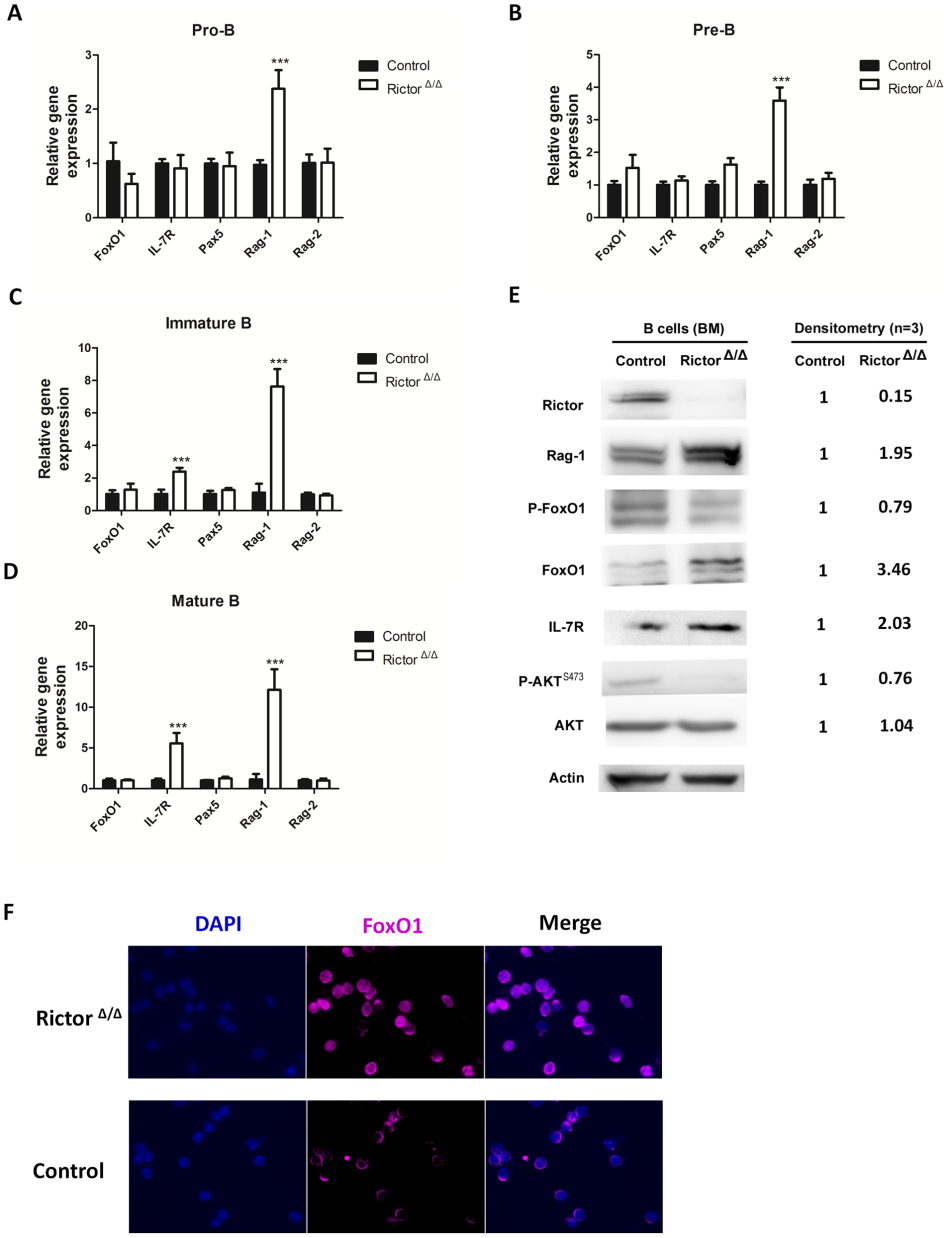
Rictor-deleted B cells exhibited increased expression of FoxO1 and Rag-1. (A), (B), (C), and (D) The relative expression of the indicated genes in pre-B, pro-B, immature B, and mature B cells. These cells were sorted from the BM of control and Rictor-deleted mice. The mRNA level was analyzed by real-time PCR. The data shown were normalized to GAPDH. (E) B cells from the BM of control and Rictor-deleted mice were sorted with MACS and were then lysed for Western blotting analysis. The expression levels of Rictor, FoxO1, P-FoxO1, Rag-1, IL-7R, Akt, P-Akt in the Rictor deleted B cells were normalized to Actin and are presented as the fold increase relative to these factors in the control B cells. (F) B cells from the BM of control and Rictor-deleted mice were sorted with MACS for immunofluorescence assay. Nuclei were detected with DAPI staining. FoxO1 was detected using FoxO1 antibody and Alexa Fluor647 Donkey Anti-Rabbit IgG staining. All of these experiments were performed in triplicate (*: P<0.05; **: P<0.01; ***: P<0.001).

FoxO1, which is downstream of the PI3K/Akt signaling pathway, plays an important role in B-cell development [16]. Previous results have shown that mTORC2 regulates the phosphorylation of FoxO1 by activating Akt [27]. FoxO1 functions with E2A and EBF1 to induce the transcription of Pax5, Rag-1, Rag-2, and IL-7R to drive B cell commitment. To explore whether these genes were involved in the B cell reduction induced by *Rictor* deletion, we sorted pro-B, pre-B, immature B and mature B cells from the BM of control and Rictor-deleted mice to analyze their gene and protein expression changes. Real-time PCR experiments showed that the transcription levels of *FoxO1* in all BM B cells in the control mice were similar to those in the *Rictor*-deleted mice. However, the mRNA expression of *Rag-1* gradually increased with B cell commitment in the *Rictor*-deleted mice compared with the control mice, whereas *IL-7R* expression was enhanced in the immature B cells and mature B cells in the *Rictor*-deleted mice compared with the control mice (Figure 5A–D). FoxO1 is sequestered in the cytoplasm for degradation upon phosphorylation by Akt. We speculated that *Rictor* deletion might impair the phosphorylation level of FoxO1 and/or the expression of FoxO1 at the protein level. To examine this possibility, we isolated B cells from the BM of control and *Rictor*-deleted mice using magnetic activated cell sorting (MACS) for Western blotting analysis and immunofluorescence assay. We found that the protein level of FoxO1 was significantly higher in the B cells of the Rictor-deleted mice than in the control mice, while the phosphorylation level of FoxO1 was significantly lower in the B cells of the *Rictor*-deleted mice than in those of the control mice (Figure 5E). We also found that there was more FoxO1 in the nuclei of Rictor-deleted B cells compared with the control cells (Figure 5F). We further confirmed that *Rictor* was completely deleted and that Akt phosphorylation at Ser473 was significantly decreased in the B cells from the *Rictor*-deleted mice at the protein level (Figure 5E). We also found that the protein expression of Rag-1 and IL-7R was significantly increased in *Rictor*-deleted B cells. All of the blots were analyzed and quantified with Image J (Figure 5E). Given the important roles of FoxO1, Rag-1, and IL-7R in B cell development, we speculated that an aberrant increase inFoxO1 followed by the upregulation of Rag-1 and IL-7R might lead to the impaired B cell development in *Rictor*-deleted mice.

**Figure 6:**
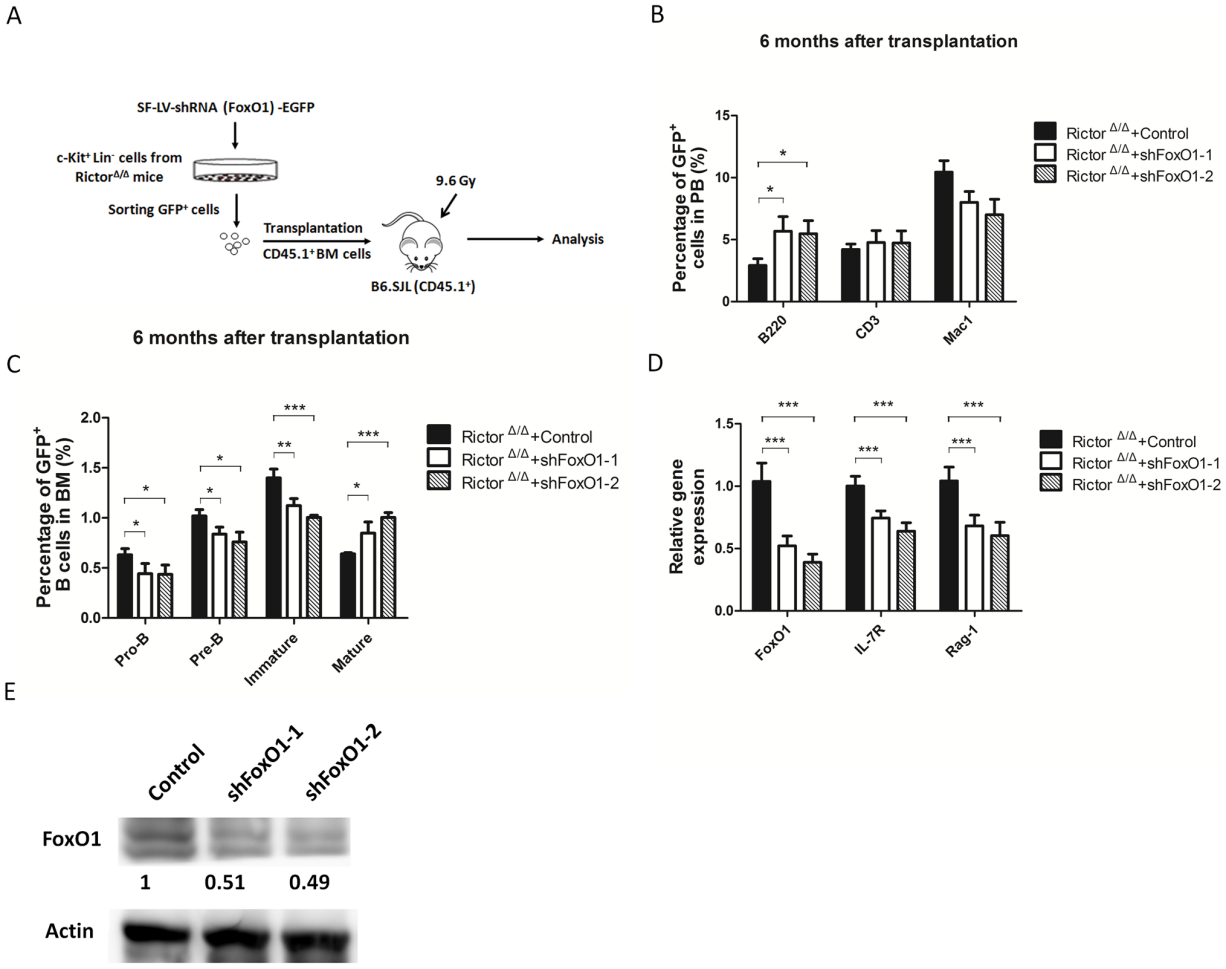
Knockdown of FoxO1 in Rictor-deficient HSPCs increased the number of B cells in PB and BM. (A) Diagram of the experimental design. *Rictor*-deficient HSPCs transduced by FoxO1 and control shRNA (GFP^+^) were transplanted into recipient mice. (B) The percentages of T, B, and myeloid cells derived from the control and FoxO1 knockdown cells (GFP^+^) in PB were analyzed by flow cytometry (n = 5). (C) The percentage of committed B cells derived from control and FoxO1 knockdown cells (GFP^+^) was detected by flow cytometry (n = 5). (D) The GFP^+^ B cells from the BM of recipients were sorted and lysed for RNA extraction. The expression of FoxO1, IL-7R, and Rag-1 at the mRNA level was analyzed by real-time PCR. (E) The GFP^+^ B cells from the BM of recipients were sorted and lysed for Western blotting. The expression levels of Rictor in the FoxO1 knockdown GFP^+^ B cells were normalized to Actin and are presented as the fold increase relative to that of the control GFP^+^ B cells. All of these experiments were performed in duplicate (*: P<0.05, **: P<0.01, ***: P<0.001).

To further examine the role of FoxO1 in the B cell development blockage induced by Rictor deficiency, we investigated whether FoxO1 knockdown could rescue the defect in the *Rictor*-deleted B cell development in BM. As shown in Figure 6A, we isolated hematopoietic stem and progenitor cells (HSPCs: Lin^−^c-Kit^+^) from *Rictor*-deleted mice and transduced them with GFP^+^ FoxO1 shRNA lentivirus; we then transplanted GFP^+^ cells with CD45.1^+^ protective cells into irradiated CD45.1^+^ recipient mice. Six months after transplantation, GFP^+^ cells comprised approximately 20% of the cells in PB and BM in the mice that received control shRNA, shFoxO1-1, and shFoxO1-2 (data not shown). The percentage of GFP^+^ B cells in the PB of shFoxO1-1 and shFoxO1-2 recipient mice was significantly higher than that in the control mice (Figure 6B). The percentages of GFP^+^ pro-B, pre-B, and immature B cells in BM of shFoxO1-1 and shFoxO1-2 recipient mice were all lower than those of the control mice, whereas the percentage of GFP^+^ mature B cells in the BM of shFoxO1-1 and shFoxO1-2 recipient mice was significantly increased compared with that of the control mice (Figure 6C). We then examined the effect of FoxO1 knockdown and Rag-1 and IL-7R expression at the mRNA level in GFP^+^ B cells from the BM of recipient mice. The expression of FoxO1 was reduced to approximately 40% and 50% at mRNA and protein level in the shFoxO1-1 and shFoxO1-2 knockdown B cells compared with the control B cells, and the mRNA expression of *Rag-1 and IL-7R* was also decreased in shFoxO1-1 and shFoxO1-2 knockdown B cells (Figure 6D and 6E). These results suggest that FoxO1 knockdown in *Rictor*-deleted HSPCs may partially rescue the defect in the *Rictor*-deleted B cell development and indicate that Rictor regulates B cell development by repressing FoxO1 and downstream Rag-1 and IL-7R.

### Rapamycin treatment aggravated the defect in B cells induced by Rictor deletion

**Figure 7:**
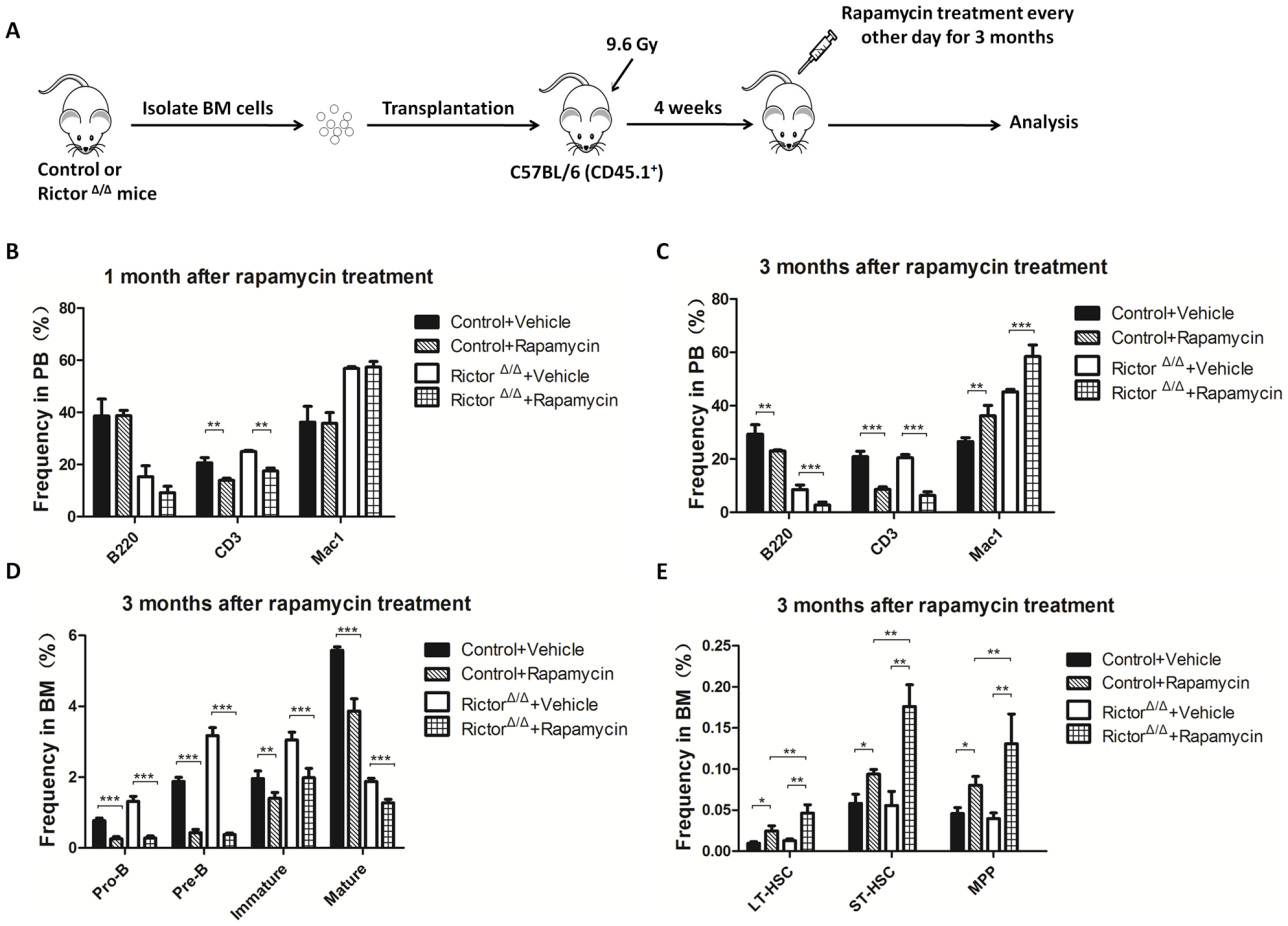
Rapamycin treatment aggravated the Rictor deletion-induced defect in B cell development. (A) A schematic diagram of the experimental design. (B) and (C) The percentage of multilineage cells in PB was analyzed by flow cytometry after vehicle or rapamycin treatment for 1 and 3 months (n = 7). (D) The percentage of committed B cells in BM after vehicle or rapamycin treatment for 3 months (n = 5). (E) The percentage of hematopoietic stem and progenitor cells after vehicle or rapamycin treatment for 3 months (n = 5). All of these experiments were performed in duplicate (*: P<0.05, **: P<0.01, ***: P<0.001).

mTOR has been shown to participate in two signaling complexes with distinct cellular functions: mTORC1 and mTORC2 [18]. Our results demonstrated that the disassembly of mTORC2 caused by *Rictor* deletion blocked the development of B cells. We were interested in whether rapamycin could inhibit the defect in B cell development in *Rictor*-deleted mice. Therefore, we transplanted BMMNCs from *Rictor*-deleted or control mice into recipient mice. One month after transplantation, the recipient mice were treated with rapamycin (Figure 7A), and the percentage of donor-derived cells in the PB was examined one and three months after the rapamycin treatment. The results showed that T cells, but not B cells and myeloid cells, were impaired one month after rapamycin treatment in the control mice and the *Rictor*-deleted mice. Three months after the rapamycin treatment, the percentages of B cells and T cells were significantly decreased, whereas the percentage of myeloid cells was increased in the control and *Rictor*-deleted BMMNC-transplanted mice (Figure 7C). Subsequently, we examined whether rapamycin treatment affected early B cell development after transplantation into recipient mice. The results showed that rapamycin treatment dramatically reduced the quantity and percentage of pro-B, pre-B, immature B, and mature B cells in the control and *Rictor*-deleted BMMNC-transplanted mice (Figure 7D). We also found that the percentage of B cells in the PB and BM of the Rictor-deleted BMMNC-transplanted mice was significantly lower than that of the control BMMNC-transplanted mice after rapamycin treatment (Figures 7C and 7D). This result indicated that rapamycin treatment further aggravated the B cell development defect induced by the Rictor deletion. We further examined the effect of rapamycin treatment on hematopoietic stem cells and progenitor cells derived from control or *Rictor*-deleted mice. The results showed that the percentages of LT-HSC, ST-HSC, and MPP all increased after rapamycin treatment in the control and *Rictor*-deleted BMMNC-transplanted mice (Figure 7E).

## Discussion

mTOR has been widely implicated in the regulation of key processes in hematopoiesis [28], [29]. *Rictor* deletion prevents leukemogenesis and HSC depletion after *Pten* deletion in adult mice, demonstrating that mTORC2 activation participates in these processes, although a *Rictor* deletion had a minimal effect on the function of normal HSCs [23]. Here, we observed that *Rictor* deletion did not impair the frequency and function of HSCs (Figure 2B and Figure 3D), suggesting that mTORC2 is not involved in the maintenance and differentiation of HSCs. *Rictor* deletion also did not lead to the development of leukemia after treatment with pIpC for approximately 2 years (data not shown), indicating that *Rictor* deficiency alone is not sufficient to initiate leukemogenesis. Nevertheless, Mark Boothby and his colleagues reported that *Rictor* deletion lowered the CCR7 expression in thymocytes and leukemic cells and that this change was accompanied by decreased tissue invasion and delayed mortality in T-ALL driven by Notch. These researchers further demonstrated that *Rictor* deletion impaired the Notch-driven proliferation and differentiation of pre-T cells [30]. Another study demonstrated that the targeted mutation of *Rictor* in thymocytes drastically reduced thymic cellularity and that Rictor deficiency caused a partial block of thymocyte development at the double-negative 3 stage [31]. We also found that *Rictor* deletion dramatically reduced the weight of the thymus and the number of thymocytes in the thymus (data not shown), although the decrease in the number of T cells in the PB was not significant compared with the control (Figure 1).

In our study, we focused on the development of early B cells in BM. Using *Rictor* conditional knockout mice, we found that *Rictor* deletion significantly increased the number of pro-B, pre-B, and immature B cells and decreased the numbers of mature B cells in BM (Figure 2). In the Rictor-deleted HSC-transplant recipient mice, early B cell development in BM was impaired, as it was in the *Rictor*-deleted mice (Figure 3), demonstrating that the B cell development defect in *Rictor*-deleted mice is independent of the hematopoietic environment and that Rictor regulates early B cell development in a cell-intrinsic manner. We observed that *Rictor* deletion impaired the cell cycle status of mature B cells and promoted the progression of more mature B cells into the G1 and S/G2/M phases, although this finding did not explain why *Rictor* deficiency caused the decrease in mature B cells in BM (Figure 4). FoxO1 and Rag-1 are the key regulators of B cell development. We found that *Rictor* deletion induced an increase in FoxO1 at the protein level, but not at the mRNA level (Figure 5). This result is consistent with a previous report indicating that Akt regulates FoxO1 at the post-transcription level by phosphorylating FoxO1 and sequestering FoxO1 in the cytoplasm for degradation [32], [33]. Rag-1, one of the downstream targets of FoxO1, mediates V-DJ recombination to generate BCR [15], [34]. We observed that *Rictor* deletion induced an increase in Rag-1 at the mRNA and protein levels in B cells (Figure 5). Furthermore, the knockdown of FoxO1 in *Rictor*-deleted HSPCs reduced the percentage of Pro-B, Pre-B, and immature B cells while increased the percentage of mature B cells in the BM of recipient mice (Figure 6). This result indicated that the knockdown of FoxO1 may partially rescue the B cell development defect induced by *Rictor* deletion. These results suggest that Rictor regulates early B cell development in BM by suppressing FoxO1 and Rag-1 in a cell-intrinsic manner. Similar to our results, Lazorchak and his colleagues found that Sin1, another component of mTORC2, regulates B cell development [35]. They used Sin1 knockout fetal liver hematopoietic cells to perform BM transplantation to study the role of Sin1 in B cell development, whereas we used *Rictor* conditional knockout mice to study the role of B cells in early B cell development. Moreover, they cultured Sin1 knockout pre-B cells *in vitro* to examine the expression of FoxO1, IL-7R, Rag-1 and other related genes, whereas we isolated primary B cells from *Rictor*-deleted mice to analyze the expression of FoxO1, IL-7R, Rag-1 and other related genes at the mRNA and protein levels that are more pertinent to *in vivo* changes in *Rictor*-deficient mice. In addition to their findings, we found that FoxO1 knockdown in *Rictor*-deleted HSPCs may partially rescued the B cell development defect in recipient mice. Nevertheless, by deleting different components of mTORC2, *Rictor*, and *Sin1*, our present study and the work of Lazorchak et al. demonstrated that mTORC2 plays an essential role in early B cell development in BM. Recently, Keunwook Lee and his colleagues reported that Rictor is required for the homeostasis and function of peripheral mature B cells by regulating the NF-κB signaling pathway [36]. This finding is consistent with our results showing that the absence of *Rictor* dramatically decreases the numbers of B cells in the PB and the spleen (Figure 1). In contrast with their study, we focused on Rictor's role in early B cell development in BM, and we demonstrated that mTORC2 not only regulated the B cell maintenance in spleen but also played a key role in early B cell development in BM.

mTOR is the catalytic subunit of mTORC1 and mTORC2. Using a *Rictor* conditional knockout, we studied the role of mTORC2 in hematopoiesis. To further elucidate the role of mTOR in hematopoiesis, we used rapamycin to treat recipient mice that were transplanted with Rictor-deleted BMMNCs. Previous studies established that rapamycin has profound effects on B cell proliferation and differentiation [37], [38], [39]. However, rapamycin does not acutely inhibit mTORC2 activity; however, chronic treatment with rapamycin can suppress mTORC2 assembly in some cell types [40]. Our study demonstrated that rapamycin treatment aggravated the mTORC2 depletion-induced decrease in immature B cells in BM. In addition, compared with vehicle treatment, rapamycin treatment reduced the number of normal pro-B, pre-B, immature B, and mature B cells in the control mice. These results indicate that both mTORC1 and mTORC2 regulate the development of early B cells (Figure 7). Consistent with previously published results [22], we also found that mTORC1 inhibition increased the frequency of HSCs (Figure 7). Moreover, rapamycin treatment increased the frequency of *Rictor*-deleted HSCs, suggesting that the increase in HSCs caused by mTORC1 inhibition was independent of mTORC2.

Taken together, the results of this study indicate that mTORC2 regulates the development of early B cells and their differentiation in a cell-intrinsic manner. We provide additional evidence that FoxO1 and downstream Rag-1 and IL-7R are regulated by Akt. Thus, our findings provide a greater understanding of the mechanism of mTORC2 and the PI3K/Akt signaling pathway in B cell development and differentiation. Exploration of the activity regulation differencesbetween mTORC1 and mTORC2 might lead to new therapeutic interventions for hematological cancer.
